# Nyphomania and Vaginismus Caused by Anal Fissure

**Published:** 1875-04-15

**Authors:** 


### NYMPHOMANIA AND VAGINISMUS CAUSED BY ANAL FISSURE.

*From the Detroit Review.*

IN a recent paper "On some Forms of Rectal Diseases of Women," Dr. Edward W. Jenks, of Detroit, writes as follows:

Many of the disorders to which the rectum is subject are, without doubt, of much more frequent occurrence among women than men. The association of uterine and rectal diseases is quite common, as may also be said of uterine and vesical disorders. The truth is, that the continuity of tissues, the distribution of nerves and vessels, and the intimate relationship existing between the pelvic organs, render it obvious why disorder either of function, structure, or place of one may produce analogous condition in another. To treat symptoms alone in diseases of the pelvic region, as in any other part of the body where viscera can be subjected to physical exploration, is most thoroughly unscientific. There are certain symptoms common to all pelvic diseases of women; others are peculiar to certain individual diseases. I am prompted to mention this as prefatory to a short paper upon certain forms of rectal diseases, for the reason that I have known many cases of disorders of the rectum where the patient had been treated alone for uterine disease, and *vice versa*, and I design mentioning some cases in illustration of the assertion.

[From those reported, we extract the following interesting cases of Fissure of the Anus:]

*Nymphomania.*—M. R., aged 20, unmarried, was sent to me by one of my former pupils as a patient that was extremely unfortunate, on account of her insatiable venereal appetite, but that it had existed only a short time, and that as yet she had not been guilty of any offence in that direction beyond masturbation. She was put in a quiet boarding-house, and under the most careful surveillance. Every symptom indicated some pelvic disorder, rather than a mental disease. Examination of the generative organs revealed no indication of their disease beyond an undue congestion of the external organs, and especially in the neighborhood of the clitoris, where there was a slight abrasion. I learned that defecation was followed by excruciating pain, and upon examining the rectum I found a broad, irritable ulcer, just within the verge of the anus. Ether was administered by Dr. Walker, when I made forcible dilatation of the *sphincter ani externum*, after the manner first suggested by Recamier. It is needless for my present purpose to follow up the details of treatment in this and the other cases reported in this paper. I will only add that it was a matter of astonishment to the few medical gentlemen familiar with the history of this patient to observe that, with the cure of the anal fissure, the nymphomania disappeared. She has since married, and when I saw her last she informed me that she was in excellent health, and happy in her new relationship.

*Vaginimus.*—Mrs. S., aged 34, had considered herself for some years as a sufferer from uterine disease. When I first saw her she was confined to her bed, and had been most of the time for the preceding six months. She had been told by her medical attendant that in all probability there was some disorder of the womb that was producing all her physical ailments. The local treatment advised consisted of emollient and astringent vaginal washes. Every symptom of which I could learn caused me to think there was some form of disease of the uterus or its annexes. Upon attempting to make a digital examination of generative organs, I was compelled to desist on account of the severe spasmodic pain my finger produced at the ostium vaginæ. My diagnosis of the case was vaginismus, which was further corroborated when

I learned that coitus had been for a long time impossible, and the few attempts at it had been followed by days of suffering. At this time my attention was not directed to the rectum for any symptom or complaint of the patient, but at a subsequent visit I found her exhibiting symptoms of the most intense agony, and upon inquiry as to its cause learned that her bowels had just moved, and that for more than two years, whenever she had had an evacuation of the bowels, her sufferings had been of a like character, while for the past few months they had been gradually increasing in severity.

I then attempted to make a digital examination of the rectum, but soon found that it caused such severe pain that I deferred it two days, when ether was administered. I then discovered the cause of the patient's intense suffering to be due to a broad and unusually deep fissure within the anus, together with one of those small and extremely sensitive excresences so frequently found cropping out from the mucous membranes of this locality, as associates of irritable ulcers of the rectum. I removed the small growth, and then forcibly dilated the sphincter after the usual method. The patient made a good recovery, and with the cure of the fissure there was an end to both the painful defecation and the vaginismus. The removal of the latter difficulty was apparently quite as satisfactory as the relief from physical suffering, for its existence had begun to be a cause of the most disagreeable sort of domestic unhappiness. The transition from a bedridden patient to a lady of active habits was quite rapid, nor did she require much further medical treatment.

---

THE PATHOLOGY OF THE BLOOD.—M. Laptschinsky, of St. Petersburg, contributes a paper to the *Centralblatt*, on the microscopic changes undergone by the blood in various diseases. He finds that in various diseases in which marked febrile symptoms are present, the microscopic aspect of the blood is essentially different from that of health. The changes consist in the blood-corpuscles not running into regularly formed rouleaux, but accumulating in heaps or clumps of various size and shape. The individual blood-corpuscles frequently appear swollen and cloudy, and their contours less distinct than natural. Small corpuscles, one-third of the normal size, are often met with, some of which exhibit a more intense color than natural, whilst others are completely pale. In the interspaces of the clumps of red corpuscles, great numbers of white corpuscles may be seen, often coalescing to form groups of from 3 to 8. In typhus he counted from 60 to 80, and more, in one field of vision; in cholera from 110 to 130. Careful enumeration of the relative numbers of white and red corpuscles four days after death in the above cases showed that there was 1 white to 60 red corpuscles in the case of typhus, and 1 white to 23 colored in the case of cholera. In a very anæmic woman, suffering from suppuration in the knee-joint, the proportion of the white rose to 1 to 13 red. The white corpuscles in these cases presented unusually active and extensive amœboid movements. The nuclei of the colorless corpuscles took a part in the amœboid movements, and could be seen altering their position and form in the interior of the white corpuscles. The thorn-apple or horse-chestnut-like form of the red corpuscles he did not find to be unusually frequent. He found, however, large quantities of granular or detritus-like material in the blood of febrile, but not much in the blood of cachectic and anæmic patients. From his enumerations he feels satisfied that in febrile diseases, and in Bright's disease, the conversion or development of white corpuscles into red is either materially retarded, or is entirely arrested.—*London Lancet*, Jan., 1875.

PHOSPHIDE OF ZINC.—At a recent meeting of the Obstetrical Society (*Lancet*, Feb. 27) Mr. J. Ashburton Thompson communicated a short paper exemplifying the advantage of employing phosphide of zinc in the treatment of chlorosis and anæmia. It succeeded in relieving the symptoms where iron had failed, and that rapidly. Phosphorus was of great value in the treatment of patients recovering from uterine hæmorrhage, and in all cases of anæmia, and seemed to exercise a specific influence upon the neuralgia so often met with in these cases, encouraging the general nutrition of the body. Free phosphorus is not the treacherous poison it has hitherto been considered to be. It is a fatal and potent poison, it is true, but its therapeutic effects may be obtained with precision and perfect safety. If proper formula be employed, no apprehension of unexpected or uncontrollable poisonous effects of a therapeutic dose need hinder its general employment.

Dr. Routh said it might be known to some members of the Society that he had made several inquiries on the employment of phosphorus. In some cases it produced marvels, especially in cases like those detailed by Mr. Thompson, due to deficiency of phosphorus in the system. But it occasionally acted injuriously, by producing headache and giddiness; and in a few cases (of idiosyncrasy perhaps) it acted as a deadly poison, even in the first dose, sometimes immediately producing vomiting and syncope. The safest preparation was the phosphide of zinc; but it was a medicine always to be closely watched when given.

Dr. Tilt inquired what preparation of phosphorus Mr. Thompson recommended.

Mr. Thompson, in reply, said Dr. Routh had revived the old objection, not because it was a poison, but because we cannot calculate the results. These arose from decomposed phosphorus.

Dr. Playfair inquired if the zinc phosphide was not insoluble in pill.

Mr. Thompson replied that some acid tonic given simultaneously would serve to assist in the decomposition. —*Phil. Med. Times.*

---

SALICYLIC ACID.—The *Boston Medical and Surgical Journal*, March 11, contains an abstract of a letter received by Prof. Horsford from Kolbe, of Leipsic, in which the latter gives various details of interest relative to the use of this product. In the lying-in hospital of Leipsic it seems to have superseded carbolic acid as a disinfectant for the hands, vaginal douching, application to ulcera puerperalia, etc. It is used in solution in water of one part in three hundred to one part in nine hundred, or as a powder mixed with starch in proportion of one part in five. Kolbe recommends the trial of this remedy internally in the various zymotic diseases, in pyæmia, bites of dogs, etc. In order to prove its innocuousness, he administered to himself and a number of students various doses of the acid, up to twenty-three grains a day. The following is recommended by Wunderlich as a convenient formula for its administration:

℞ Acidi salicylici, gr. xvi;

Ol. amygdalæ dulc, f ʒ vi;

Acaciæ gummi, ʒ iii;

Syrupi amygdalæ, f ʒ viiss;

Aq. aurantii flor., ad f ℥ iv.—M.

Salicylic acid is very little, if at all, absorbed through the skin.—*Phil. Med. Times.*

---

IODINE AS A SUBSTITUTE FOR IODIDE OF POTASSIUM.—It is found that in some cases, where iodide of potassium is not tolerated by the stomach, iodine itself can be administered in the form of pills.

The iodine is first suspended in albumen, and afterward the combination is dried and powdered. In this way one-quarter grain of iodine can be made into a pill, and two and a half grains can be given daily, without disordering the stomach. This method was obtained from Paris, and proves of much service in relieving tertiary symptoms.—*N. Y. Medical Journal.*

ON THE EFFECT OF VENOUS OBSTRUCTION OF THE SKIN.—Dr. Auspitz has lately brought before the k. k. Gesellschaft der Aerzte in Vienna, some observations upon the effect of venous obstruction of the skin, which are reported in the *Allgemeine Wiener Medizin. Zeitung* for November 3.

1. A ligature was tied round the arm of a healthy person, as if for bleeding. First the superficial veins swelled, then a livid color spread over the arm, beginning on the flexor surface (where the skin is thinnest), and at last affecting all but the volar eminence (the thickest part of the skin). At the same time the temperature sank. The next effect was œdema of the skin, followed in from five to ten minutes by the appearance of numerous patches of red or brownish-red color, accompanied by minute spots, which were either bright scarlet or purple in tint. On removing the bandage, the cyanosis first disappeared, then the œdematous swelling; next the red patches gave place to a diffused blush of the whole arm; and lastly this disappeared, leaving only the minute red spots, which remained for several hours or even days. Comparing these appearances with those observed by Cohnheim in a rabbit's ear as the result of mechanical venous obstruction, there can be no doubt that the minute red spots above mentioned are extravasations, either of red blood-corpuscles or of their hæmoglobin in solution.

2. Similar experiments were next tried by Dr. Auspitz on the arms of persons suffering from measles, variola, and other cutaneous diseases.

*a.* In the cases of measles, it was observed that the large red patches above mentioned coincided with those of the eruption; and that beside the minute ecchymoses, larger spots of cutaneous hæmorrhage sometimes appeared.

*b.* The effect in cases of urticaria was less marked. The wheals were more prominent, and minute ecchymoses were not more frequent (perhaps less so) than in the normal skin.

*c.* In ordinary small-pox, there was intense congestion of the bases of the pustules, and the points of cutaneous hæmorrhage were both more numerous and larger than in a normal arm; but there was never any extravasation of blood in the pustules themselves.

*d.* In cases of hæmorrhagic variola, the whole of the arm below the ligature became rapidly covered by a dark-blue lividity, which concealed all minor shades of color. This was the case even when there were few or no pustules; and when these were present, they were not themselves the seat of extravasation of blood.

*e.* The ligature applied to the arm of patients suffering from scarlatina, had little or no effect beyond that observed in the experiments above noted on the normal skin.

*f.* In "scorbutic affections—*erythema nodosum*, *morbus maculosus Werlhoffii*, *purpura rheumatica*, and scurvy proper"—Dr. Auspitz was surprised to find the effect of the ligature trifling. There was no scarlet injection of the skin and no ecchymosis. This seems to confirm the old belief (recently called in question by Cohnheim) that purpura depends on a change in the blood itself, and not on any difference of pressure in the circulation, or on anatomical lesions of the blood-vessels.—*London Medical Record*, December 30, 1874.

PRACTICE FOR SALE.

A Physician's practice, good will, etc., at one of the famous Springs, South. Price, $20,000 cash, or good secured paper. The practice now amounting to upwards of $40,000 actual cash receipts yearly, and increasing. The Physician who wishes to sell, has amassed a competency, and wishes to retire. For full particulars, please address, L. A. GILBERT,
206 La Salle Street,
Chicago, Ill.

ANATOMICAL PREPARATIONS,

Including Skeletons, and the Separate Bones of the Human Subject, can be obtained at reasonable prices, of
E. H. SARGENT,
Dealer in Surgical Instruments,
CHICAGO.

